# Donepezil Does Not Enhance Perceptual Learning in Adults with Amblyopia: A Pilot Study

**DOI:** 10.3389/fnins.2017.00448

**Published:** 2017-08-07

**Authors:** Susana T. L. Chung, Roger W. Li, Michael A. Silver, Dennis M. Levi

**Affiliations:** School of Optometry, Vision Science Graduate Group, Helen Wills Neuroscience Institute, University of California, Berkeley Berkeley, CA, United States

**Keywords:** amblyopia, cholinesterase inhibitors, donepezil (aricept), perceptual learning, contrast sensitivity, uncrowding

## Abstract

Amblyopia is a developmental disorder that results in a wide range of visual deficits. One proven approach to recovering vision in adults with amblyopia is perceptual learning (PL). Recent evidence suggests that neuromodulators can enhance adult plasticity. In this pilot study, we asked whether donepezil, a cholinesterase inhibitor, enhances visual PL in adults with amblyopia. Nine adults with amblyopia were first trained on a low-contrast single-letter identification task while taking a daily dose (5 mg) of donepezil throughout training. Following 10,000 trials of training, participants showed improved contrast sensitivity for identifying single letters. However, the magnitude of improvement was no greater than, and the rate of improvement was slower than, that obtained in a previous study in which six adults with amblyopia were trained using an identical task and protocol but without donepezil (Chung et al., 2012). In addition, we measured transfer of learning effects to other tasks and found that for donepezil, the post-pre performance ratios in both a size-limited (acuity) and a spacing-limited (crowding) task were not significantly different from those found in the previous study without donepezil administration. After an interval of several weeks, six participants returned for a second course of training on identifying flanked (crowded) letters, again with concurrent donepezil administration. Although this task has previously been shown to be highly amenable to PL in adults with amblyopia (Chung et al., 2012; Hussain et al., 2012), only one observer in our study showed significant learning over 10,000 trials of training. Auxiliary experiments showed that the lack of a learning effect on this task during donepezil administration was not due to either the order of training of the two tasks or the use of a sequential training paradigm. Our results reveal that cholinergic enhancement with donepezil during training does not improve or speed up PL of single-letter identification in adults with amblyopia, and importantly, it may even halt learning and transfer related to a crowding task.

**Clinical Trial Registration:** This study was registered with ClinicalTrials.gov (NCT03109314).

## Introduction

Amblyopia is a developmental disorder of spatial vision that reflects the neural impairment that occurs when normal visual development is disrupted, and it is therefore an ideal model for understanding when and how brain plasticity may be harnessed for recovery of function. Amblyopia is one of the leading causes of vision loss in young children, affecting approximately 2–4% of the population (Ciuffreda et al., 1991).

In the clinic, amblyopia is generally treated in young children by patching or penalizing the stronger eye. However, it is rarely treated in older children or adults because it is commonly assumed that there is limited visual system plasticity after the “critical period” of visual development has ended. One approach to recovering vision in human adults with amblyopia is perceptual learning (PL), in which patients practice a challenging visual task. In most cases, the reduction in visual acuity deficits, the *sine qua non* of amblyopia, following PL is limited to about 1 to 2 lines on a visual acuity chart (Levi and Li, 2009; Tsirlin et al., 2015).

Recent studies in adult animals with amblyopia also indicate that it may be possible to restore plasticity (Bavelier et al., 2010), although brain plasticity remains more restricted in scope in adults than during development. At a cellular and molecular level, adult plasticity is limited. Some of these “brakes” on plasticity are structural, such as peri-neuronal nets or myelin, which inhibit neurite outgrowth. Others are neurochemical and affect cortical synaptic transmission (Bear and Singer, 1986; Kasamatsu, 1991; Maya Vetencourt et al., 2008; Harauzov et al., 2010; Morishita et al., 2010).

Plasticity in adulthood can be induced either by lifting these brakes through invasive interventions or by exploiting endogenous permissive plasticity factors such as neuromodulators (Bavelier et al., 2010). The plastic potential of neural networks can be altered late in life by acutely regulating excitatory and inhibitory (E/I) transmitter signaling (Bavelier et al., 2010; Morishita et al., 2010; Baroncelli et al., 2011, 2012; Sale et al., 2011). Manipulations that locally reduce inhibition in the adult brain have been found to restore plasticity to a level comparable to that seen in the developing brain (Sugiyama et al., 2008; Harauzov et al., 2010). In addition, neuromodulators such as norepinephrine, acetylcholine (ACh), serotonin, and dopamine constitute an array of potent endogenous systems for regulating adult cortical plasticity (Bear and Singer, 1986; Kasamatsu, 1991; Kilgard and Merzenich, 1998; Bao et al., 2001; Weinberger, 2007; Maya Vetencourt et al., 2008). For example, systemic administration of the acetylcholinesterase inhibitor physostigmine restores visual function in amblyopic adult mice by enhancing endogenous ACh signaling (Morishita et al., 2010). Achieving a similar result in humans would be a significant advance.

In the present study, we combined the use of a cholinesterase inhibitor (donepezil; trade name: Aricept) to increase synaptic levels of ACh in the brains of adults with amblyopia while they were sequentially trained on two PL tasks: single letter identification and flanked letter identification. Oral administration of donepezil is widely used in the treatment of Alzheimer's disease and is considered to be safe, with few side effects. Cholinesterase inhibitors like donepezil are very useful for understanding the endogenous functions of ACh, since at any given time, the drug can only have a physiological effect at a synapse that is currently releasing ACh, unlike receptor agonists and antagonists that interact directly with neurotransmitter receptors. Donepezil blocks the metabolism of ACh in the synapse, thereby prolonging its effective lifetime and presumably augmenting whatever effects ACh would normally have at that synapse. Thus, donepezil likely increases cholinergic signaling through the receptor subtypes that are normally activated by ACh.

Donepezil has been shown to enhance PL of motion direction discrimination in adults with normal vision (Rokem and Silver, 2010). Moreover, the beneficial effects of cholinergic enhancement with donepezil on PL persist for at least several months after the end of training and drug administration (Rokem and Silver, 2013). In addition, significant behavioral improvement on a three-dimensional multiple object tracking task was observed at an earlier time point when training occurred under the influence of donepezil compared to placebo (Chamoun et al., 2017). Based on these findings we hypothesized that increasing ACh levels would make PL faster and more effective in adults with amblyopia than PL alone.

In this study, we used a sequential-training design that included two phases of training. In Phase 1, observers were trained on a single-letter identification task in which the primary measurement was the contrast threshold for letter identification. In a previous study, training on this task (without donepezil) resulted in substantial improvements in contrast sensitivity that also transferred to better letter acuity and reduced crowding in adults with amblyopia (Chung et al., 2012).

A subset of the observers then underwent a second phase of training while ingesting donepezil, this time on a flanked letter identification task in which the target letter was closely flanked by two other letters. Training on this task has previously been shown to be effective in reducing the crowding extent in participants with normal vision (Chung, 2007) and with amblyopia (Chung et al., 2012). Unlike the training in Phase 1, in which performance is limited by contrast sensitivity, performance on the flanked letter identification task in Phase 2 is likely to be limited by crowding. Importantly, simply increasing synaptic ACh levels by acute administration of donepezil to adults had no effect on letter acuity or crowding in normal peripheral vision (Kosovicheva et al., 2012). Thus, the flanked letter identification task seems ideal for assessing the effects of donepezil on PL *per se* in amblyopia.

To our dismay, our results suggest that combining donepezil with PL does not result in either more or faster learning of low-contrast letter identification in adults with amblyopia, when compared with the effects of PL alone. Further, combining donepezil with learning to identify flanked letters seems to halt learning in its tracks in adults with amblyopia.

## Materials and methods

### Observers

Eleven adults with amblyopia [defined as (1) a difference in the best-corrected visual acuity between the two eyes of ≥0.2 logMAR; and (2) an acuity of 0.0 logMAR (equivalent to 20/20 Snellen acuity) or better in the non-amblyopic fellow eye], between 18 and 65 years of age (median 33.8; SD = 14.5), with strabismus (5), anisometropia (2), both (3), or congenital cataract and strabismus (1), participated in the study. Observers' visual characteristics are provided in Table 1. With the exception of control observer S11 who underwent training without taking donepezil, all other observers ingested a pill containing 5 mg of donepezil in front of the experimenter, immediately before the start of each training session. None of the observers reported any adverse side effects of taking donepezil throughout and after the study. Testing was performed using the amblyopic eye only, with the fellow non-amblyopic eye covered using a standard black eye patch. All observers wore their best optical corrections for the viewing distance during testing and training. The experimental procedures were approved by the Committee for the Protection of Human Subjects at the University of California, Berkeley. The research was conducted in accordance with principles expressed in the Declaration of Helsinki. All observers gave oral and written informed consent before the commencement of data collection.

**Table 1:** Visual characteristics of the amblyopic observers.

**Observer**	**Gender**	**Age (years)**	**Type**	**Eye**	**Refractive errors**	**Visual acuity—crowded (isolated)**		**Cover test (@ 6 m unless stated otherwise)**
						**Snellen**	**logMAR**	
**PRESENT STUDY (WITH DONEPEZIL)**								
S1	M	18.7	S	R	−6.75/−0.75 × 5	20/63^+1^ (20/50^+2^)	0.48 (0.36)	R 2^Δ^ ET
				L	−4.75/−1.00 × 150	20/16^+2^	−0.14	
S2	M	41.1	C, S	R	+0.25/−0.50 × 20	20/12^−2^	−0.16	L 6^Δ^ XT
				L	pl/−0.25 × 15 (IOL)	2/24^−1^ (2/15^−2^)	1.10 (0.92)	
S3	M	41.4	S	R	−1.25	20/63 (20/40^+2^)	0.50 (0.26)	R 25^Δ^ ET
				L	−1.00	20/16^−1^	−0.08	
S4	M	29.4	S	R	−1.25/−1.00 × 25	20/16^−1^	−0.08	L 10^Δ^ ET
				L	−1.00/−0.50 × 35	20/40^+2^ (20/20^−2^)	0.26 (0.04)	
S5	F	50.6	S	R	+1.25/−0.75 × 80	20/20^−2^	0.04	L 4^Δ^ ET
				L	+1.75/−0.75 × 100	20/32^−2^ (20/32^+2^)	0.24 (0.16)	
S6	M	22.4	A	R	+2.50/−0.50 × 80	20/40^−1^ (20/40^+2^)	0.32 (0.26)	EP
				L	−1.50	20/12.5	−0.20	
S7	F	32.1	S, A	R	−0.75/−0.50 × 140	20/16^+2^	−0.14	L 4^Δ^ XT, R 4^Δ^ HyperT (@2 m)
				L	+5.00/−1.75 × 170	1/24^−1^ (2/30^−2^)	1.40 (1.22)	
S8	F	33.8	S, A	R	−1.25	20/16^−2^	−0.06	L 35^Δ^ ET
				L	+3.00	5/100^+2^ (5/32^−1^)	1.26 (0.82)	
S9	F	47.5	S, A	R	−3.00/−2.00 × 160	20/16^+1^	−0.12	L 20^Δ^ ET, L 10^Δ^ HyperT
				L	+1.00/−1.00 × 20	20/40^+2^ (20/32^−2^)	0.26 (0.24)	
S10	M	64.6	S	R	+5.00/−0.75 × 80	20/16^+2^	−0.14	L 16^Δ^ ET
				L	+8.25/−2.25 × 75	20/100^+2^ (20/63^+2^)	0.66 (0.46)	
S11	F	17.7	A	R	+6.00/−1.00 × 165	20/40^−1^ (20/32^−2^)	0.32 (0.24)	6^Δ^ XP
				L	+2.00	20/16	−0.01	
**(Chung et al., 2012) WITHOUT DONEPEZIL**								
**SINGLE-LETTER IDENTIFICATION TRAINING GROUP**								
SP	F	22	S	R	+0.75/−1.50 × 90	20/80^−2^ (20/40^−1^)	0.64 (0.32)	10–12^Δ^ RXT
				L	−0.25	20/12	−0.20	
SDW	F	46	S	R	+2.00	20/12.5^−1^	−0.18	6^Δ^ RHyperT
				L	+3.00/−0.75 × 95	20/40^−1^ (20/25^−1^)	0.32 (0.12)	25^Δ^ LXT
PT	F	40	S	R	pl	20/12.5^+1^	−0.22	>25^Δ^ LET
				L	+1.75/−0.50 × 5	20/32^+2^ (20/25^+2^)	0.16 (0.06)	
RE	F	27	S	R	−0.50/−3.75 × 150	20/40^−1^ (20/25^−2^)	0.32 (0.14)	20−25^Δ^ RET
				L	−2.00/−3.50 × 25	20/20^−2^	0.04	
JL	M	30	A	R	−1.50/−0.25 × 160	20/16^+1^	−0.12	4^Δ^ EP
				L	+0.75/−0.75 × 170	20/63^+1^ (20/50^+2^)	0.48 (0.36)	
LA	F	47	A	R	+4.25/−4.00 × 72	20/50^−2^ (20/50^−2^)	0.44 (0.44)	Normal
				L	+0.25/−1.00 × 115	20/16^−2^	−0.06	
**FLANKED-LETTER IDENTIFICATION TRAINING GROUP**								
GDW	M	23	S	R	+3.25	20/32^+1^ (20/20^+2^)	0.18 (−0.04)	6^Δ^ RET
				L	+2.50	20/12.5^−1^	−0.18	
BP	M	67	S	R	−7.50	20/32^+2^	0.16	10^Δ^ LET
				L	−2.00/−2.25 × 5	20/400^−2^ (20/100^−2^)	1.34 (0.74)	
AS	F	32	S	R	pl/−1.00 × 120	20/63^−1^ (20/50^+2^)	0.52 (0.36)	8−10^Δ^ LET
				L	−4.00	20/16^−1^	−0.08	
JHS	F	53	S	R	+1.25/−0.50 × 150	20/16^+1^	−0.12	>30^Δ^ LXT
				L	+1.00/−0.50 × 160	20/125^−2^ (20/63)	0.84 (0.50)	
JS	F	26	A	R	+1.00	20/25^−2^ (20/25^+2^)	0.14 (0.06)	4^Δ^ EP
				L	+0.25	20/12^−2^	−0.16	

*S, Strabismic amblyopia; A, Anisometropic amblyopia; C, Congenital cataract; ET, Esotropia; XT, Exotropia; HyperT, Hypertropia; EP, Esophoria; IOL, Intraocular lens implanted*.

To calculate the number of observers required to show PL in this study, we assumed a significance level of 0.05 (two-tailed) and a power of 0.80. Our previous study (Chung et al., 2012) used essentially identical stimuli and methods for the two training tasks and obtained sizable improvement from our observers. Therefore, in this study, we estimated the expected effect size based on the main findings from that previous study—the ratios between the first and the last block of training (based on fitted curves) yielded a ratio of 0.67 ± 0.23 [mean ± 1.96 × standard error of the mean (SEM), *n* = 6] for the single letter identification training and 0.69 ± 0.15 (mean ± 1.96 × SEM, *n* = 5) for the flanked letter identification training. These values yielded a sample size of eight and four for the single and flanked letter identification training, respectively.

### Visual stimuli and psychophysical methods

The stimuli and methods were essentially identical to those used by Chung et al. (2012).

Three baseline measurements were collected before and after each phase of training, in separate blocks of trials, and always in the following order: (1) letter size limit, i.e., the smallest letter size that allowed observers to identify single letters at 52% correct [essentially 50% correct, after correction for guessing (26 possible responses)]; (2) spacing limit, i.e., the nominal center-to-center letter separation between adjacent letters such that the performance of identifying the middle letter of trigrams was 52% correct; and (3) contrast threshold for identifying single letters (see Chung et al., 2012 for details). For the spacing and contrast tests, the letter size was set to 1.5 × the letter size limit (*x*-height, defined as the height of the lowercase letter “*x*”) for each observer. We used the method of constant stimuli to test a range of letter sizes, spacings, and contrasts from which we derived psychophysical thresholds. A schematic of the visual stimuli is shown at the bottom of **Figure 5**.

Stimuli used for training were single letters or sequences of three letters (trigrams), randomly drawn with replacement from the 26 lowercase letters of the Times-Roman alphabet. Following the disappearance of the stimulus on each trial, observers reported the identity of the letters—either the single letter or the middle letter of each trigram—by typing their responses using a computer keyboard. The letter to be identified was always presented at the center of the display. Two small dots, vertically straddling this letter, were presented continuously on the monitor to aid visual fixation. Observers were asked to fixate the center between the two dots throughout testing.

There were two training tasks: single letter training and flanked letter training, each involving blocks of 100 trials. For single letter training, observers had to identify a low contrast letter that was 1.2X the pre-test letter size limit. In each block of trials, we used the method of constant stimuli to present single letters at five contrast levels (20 trials per contrast level). We fit each block of trials with a cumulative Gaussian function from which we determined the contrast threshold that yielded 52% correct letter identification. This threshold represents the performance for that block. For flanked letter training, observers identified the middle letter of a high (90%) contrast trigram, with center-to-center separation between adjacent letters fixed at 0.8X the letter size. The letter size was set to 1.5X the pre-test letter size limit. Note that for each participant, the pre-test letter size limits for single letter training and flanked letter training were determined separately, based on data from the pre-test that immediately preceded the respective training.

### Testing and training sequence

**Figure 1:**
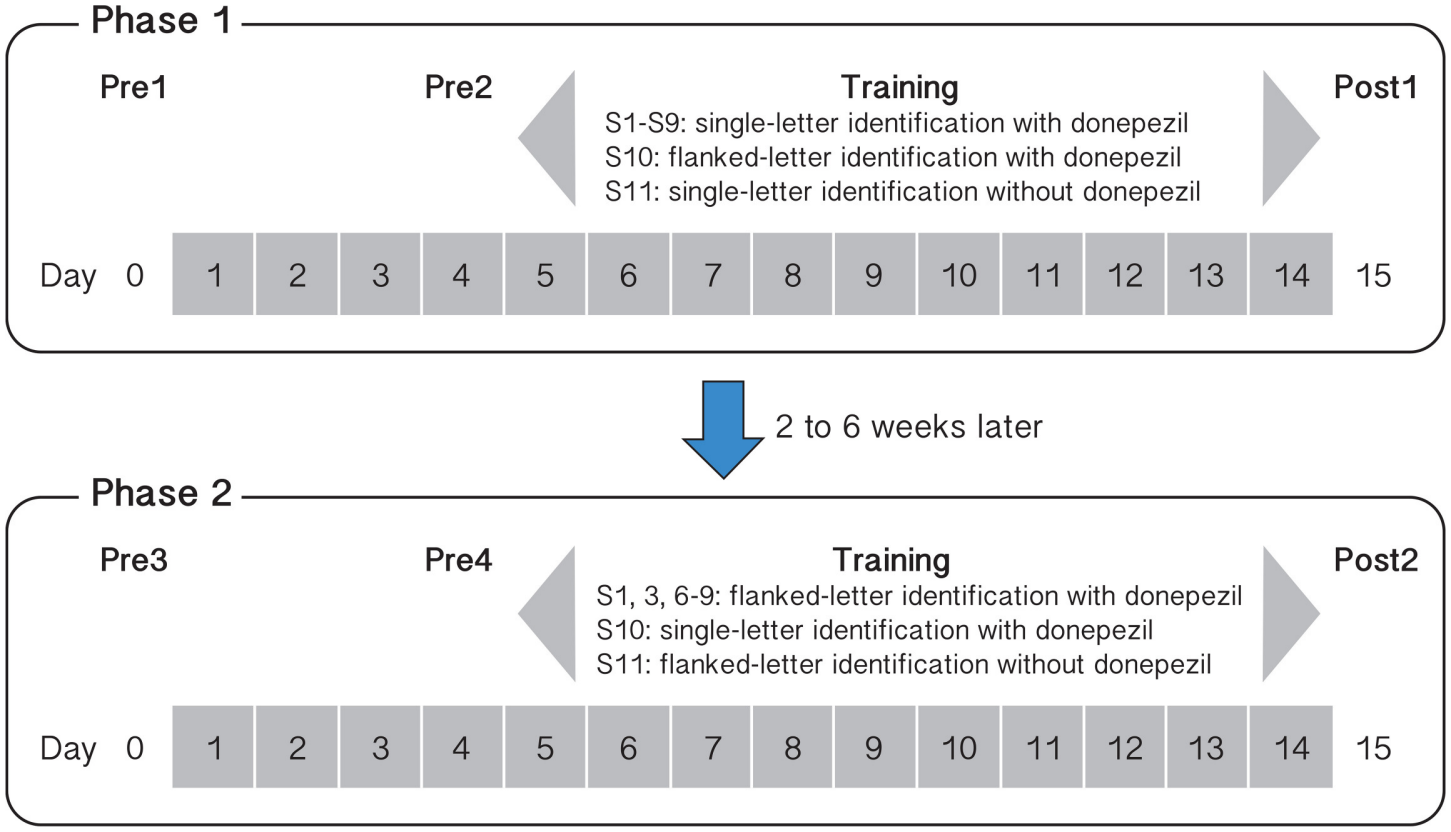
Schematic summary of the training protocol and tasks for the different observers and for the two phases of training. Day numbers within gray squares represent those days in which observers ingested 5 mg donepezil (except for observer S11 who completed the two phases of training without taking any donepezil).

Baseline measurements were made twice before each phase of training: on Day 0 (Pre1 or Pre3) and then repeated on Day 4 (Pre2 or Pre4). To ensure that training took place with steady-state plasma levels of donepezil (the half-life of donepezil in the human body is approximately 80 h, Rogers et al., 1998), we asked observers (S1–S10) to start taking daily doses of 5 mg of donepezil 3 days before training commenced. Therefore, to evaluate whether or not performance for the baseline tasks changed after training, we compared the performance between Pre2 and Post1, and between Pre4 and Post2 (1 day before and 1 day after training). Pre1 and Pre3 were sessions designed to familiarize the observers with the different tasks of the baseline measurements. Figure 1 shows the schedule of our training protocol.

Nine observers (S1–S9) underwent single letter contrast identification threshold training on days 5–14. Observers completed a total of 10 training sessions, with 10 blocks of trials (100 trials per block) per session. On day 15, they completed a post-training assessment (Post1). After an interval of at least 2 weeks (range: 2–6 weeks) following Post1, six observers began Phase 2 training while ingesting donepezil, following the same testing/training sequence as Phase 1 (see Figure 1), but the training task was changed to flanked letter identification. As with the single letter training, observers completed 10 flanked letter identification sessions on 10 consecutive days. A tenth observer (S10) performed the training in the reverse sequence—flanked letter training followed by single letter contrast identification.

## Results

We have previously shown that adults with amblyopia were able to improve on a low-contrast single letter identification task, as well as on a flanked letter task designed to reduce crowding (hence, the flanked letter task is also referred to as an “uncrowding” task), following 10 sessions (10,000 trials) of PL of the respective tasks (Chung et al., 2012). Given that these tasks relate to important characteristics of amblyopic vision (contrast sensitivity deficits and crowding, respectively) and have been shown to be effective in inducing PL in adults with amblyopia, here we used the same tasks and experimental procedures as in the previous study, with two key differences. First, in the present study, donepezil was administered during PL. Second, we used a sequential training design to assess PL for the two training tasks. Because each individual training task and the pre- and post-training baseline tests were essentially the same in Chung et al. (2012) and the present study, we analyzed data from the observers in Chung et al. (2012) as a “no donepezil control group” to assess drug effects on learning.

To quantitatively address the goal of this study (whether donepezil combined with PL is more effective than PL alone), for each training and baseline task, we compare changes in performance following training with (1) a null effect (no improvement), to establish if there is a significant improvement due to learning; and with (2) the results from Chung et al. (2012) to evaluate whether or not there is a drug effect. Unless otherwise specified, all the data met the assumption of a normal distribution, justifying the use of parametric tests to evaluate statistical significance.

### Training effects

**Figure 2:**
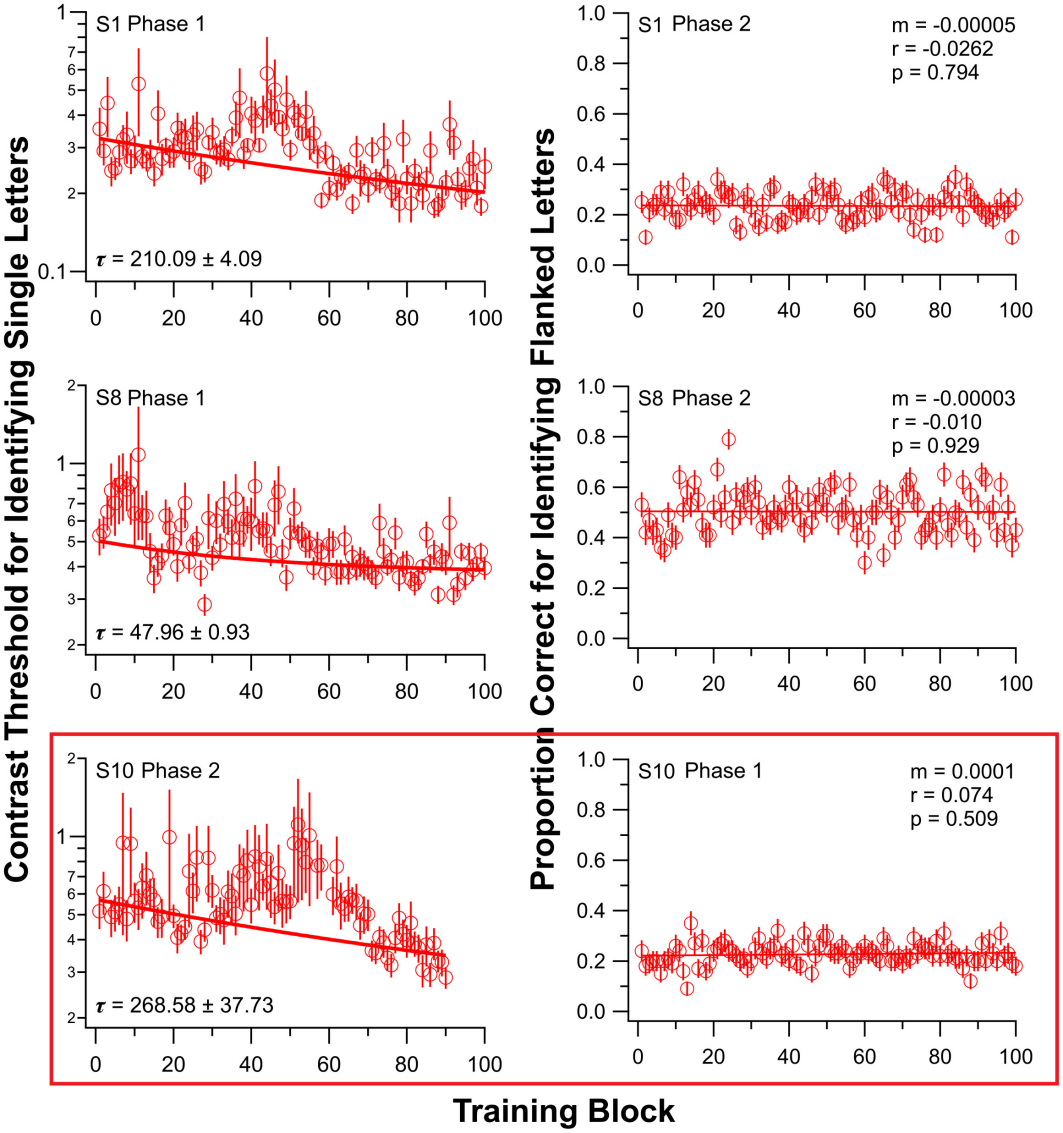
Training results from three observers, for single letter identification task **(left)** and flanked letter identification task **(right)**. Data for all other observers are presented in Figure S1. Observers S1–S9 first trained on the single letter task (Phase 1). Note that for observer S10 (bottom panels), the order of training was reversed: flanked letter identification task first (Phase 1), followed by single letter identification task (Phase 2). Due to an unfortunate incident, S10 was only trained for 9, instead of 10 sessions, in Phase 2. Error bars represent ±1 SEM.

Initially, nine observers (S1–S9) with amblyopia underwent training on the single-letter identification task. After a 2–6 weeks interval, six of these nine observers returned for training on the flanked letter training task. The other three observers were not available for additional training. Figure 2 shows the results of two of these nine observers (S1 and S8); individual observer data for the other seven observers are in Figure S1 for both training tasks. There are substantial individual differences in the learning profiles, as is typical for PL (e.g., Chung et al., 2012).

To quantify the rate of learning in individual observers, we fit each observer's single letter identification training data, weighted by the SEM, with an exponential function:

$$\begin{array}{l} y=y_{0}+Ae^{-\left(\frac{x}{\tau }\right)} \end{array}$$

In this equation, *y* is the fitted contrast threshold for block *x, y*_0_ is the fitted contrast threshold for block 0, *A* represents the amount of improvement, and τ (the decay constant) represents the rate of learning, i.e., the training block at which threshold is lowered by 37.6%. A higher value of τ means that learning is slower. We also fit individual observers' single letter identification data with a linear function, but the goodness-of-fit values (after adjusting for the different degrees of freedom) were better for the exponential fit.

To test our hypothesis that donepezil leads to faster and more substantial learning, we compared both τ and the amount of improvement (calculated as the ratio of contrast thresholds between the 100th block and the 1st block of training) for the group of observers in the present study with those from Chung et al. (2012). Individual data in Chung et al. (2012) were refit using the same exponential function described above.

**Figure 3:**
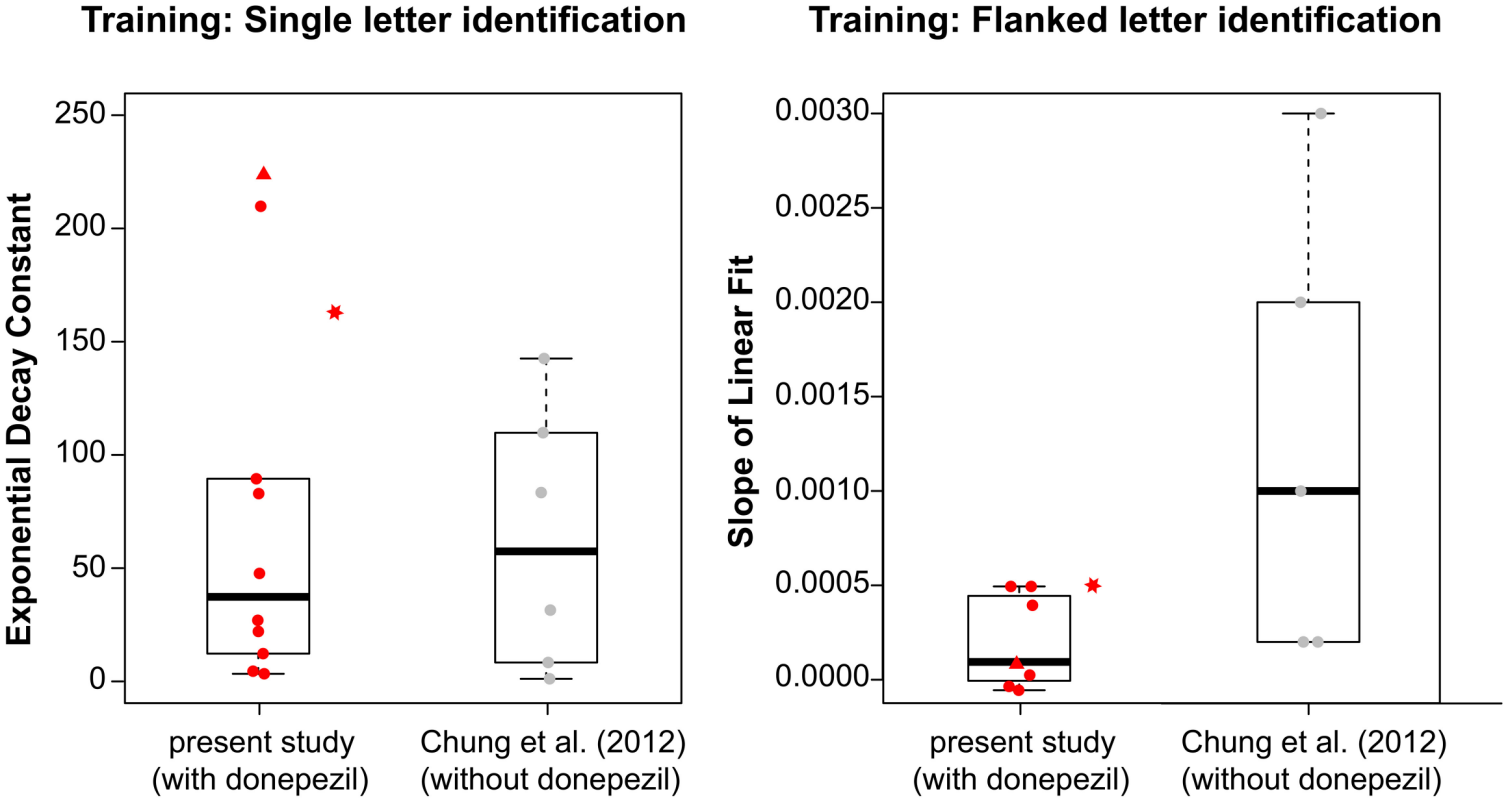
Boxplots showing the distribution of **(left)** the exponential decay constant, τ, derived from the best-fit exponential function for each observers' training data for the single letter identification training; and **(right)** the slope of the linear fit based on the best-fit line for each observer's training data for the flanked letter training, for observers with donepezil administration (present study, plotted in red) and without donepezil (Chung et al., 2012, plotted in gray). For observers with donepezil administration, circles represent observers S1–S9, triangles represent data for S10, and stars represent data for S11. The stars are plotted adjacent to the boxplot for the present study because S11 did not take donepezil during training, and thus her values were not included in the boxplot.

**Figure 4:**
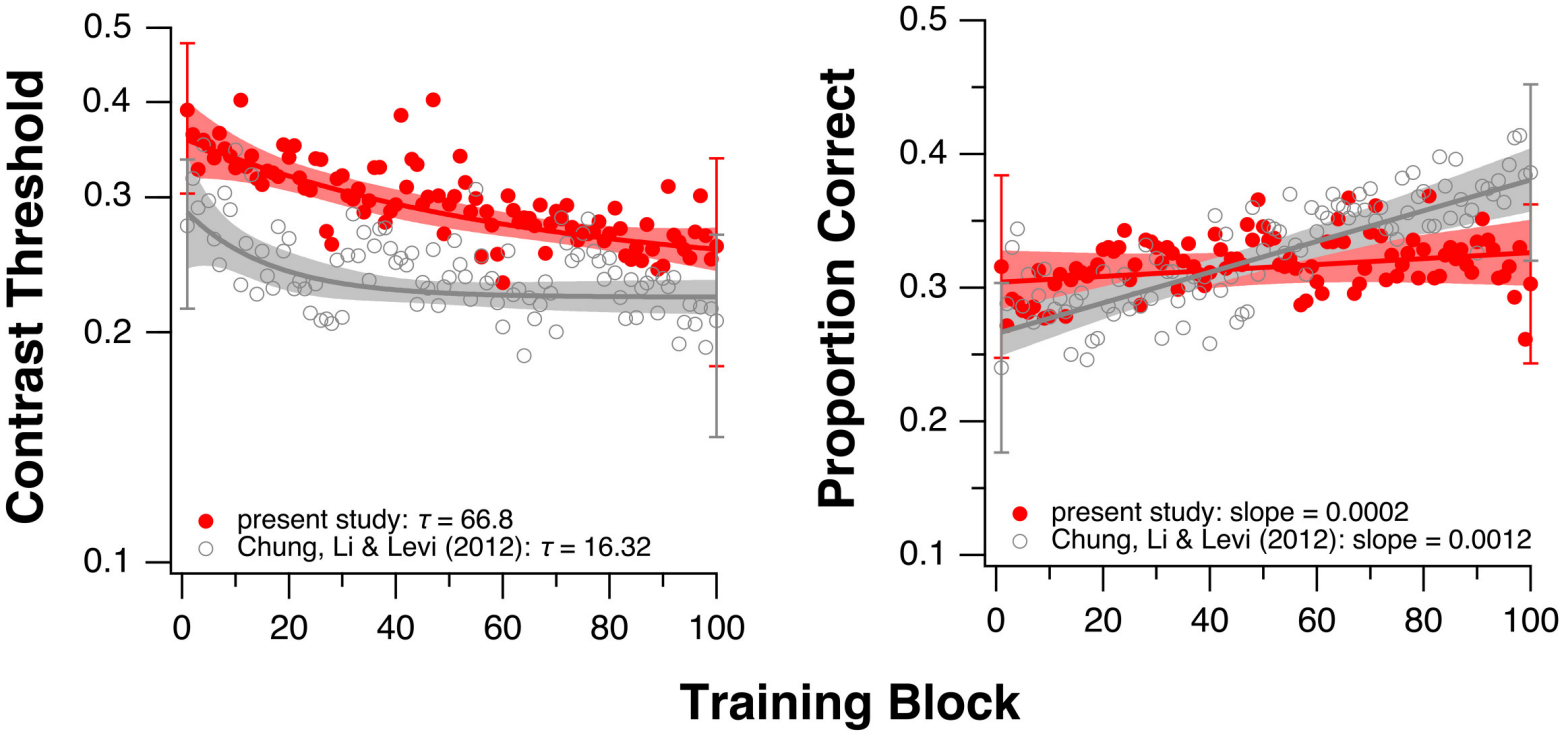
Training results averaged across all observers in the current study (red circles). Single letter identification task **(left)**. Flanked letter identification task **(right)**. For comparison, the gray circles show the averaged data from our previous study of PL without donepezil in amblyopia (Chung et al., 2012). Note that in Chung et al. (2012), instead of sequential training, the two training tasks were performed by two separate groups of observers (single letter training: *n* = 6; flanked letter training: *n* = 5). Shaded regions represent 95% confidence intervals based on the model parameter values. Error bars shown for the 1st and the 100th block represent ±1 SEM of the group-average values.

Because the fitted values of τ in the current study were not normally distributed (Shapiro-Wilk normality test: *p* = 0.0015), we used the Mann-Whitney-Wilcoxon test and found no significant difference in τ between the groups with and without donepezil (*W* = 30; *p* = 1.0). The boxplots in the left panel in Figure 3 summarize the τ-values for the two groups. This result suggests that donepezil did not affect the rate of PL, at least for the single-letter identification task. However, when we compared average data from the two groups (performance averaged across all observers of the same group—with or without donepezil) for each training block, the rate of learning appeared to be slower in the donepezil group (Figure 4, left panel; τ = 66.80 ± 2.72 [mean ± 1.96 × SEM, with donepezil] vs. 16.32 ± 0.46 [without donepezil]).

**Figure 5:**
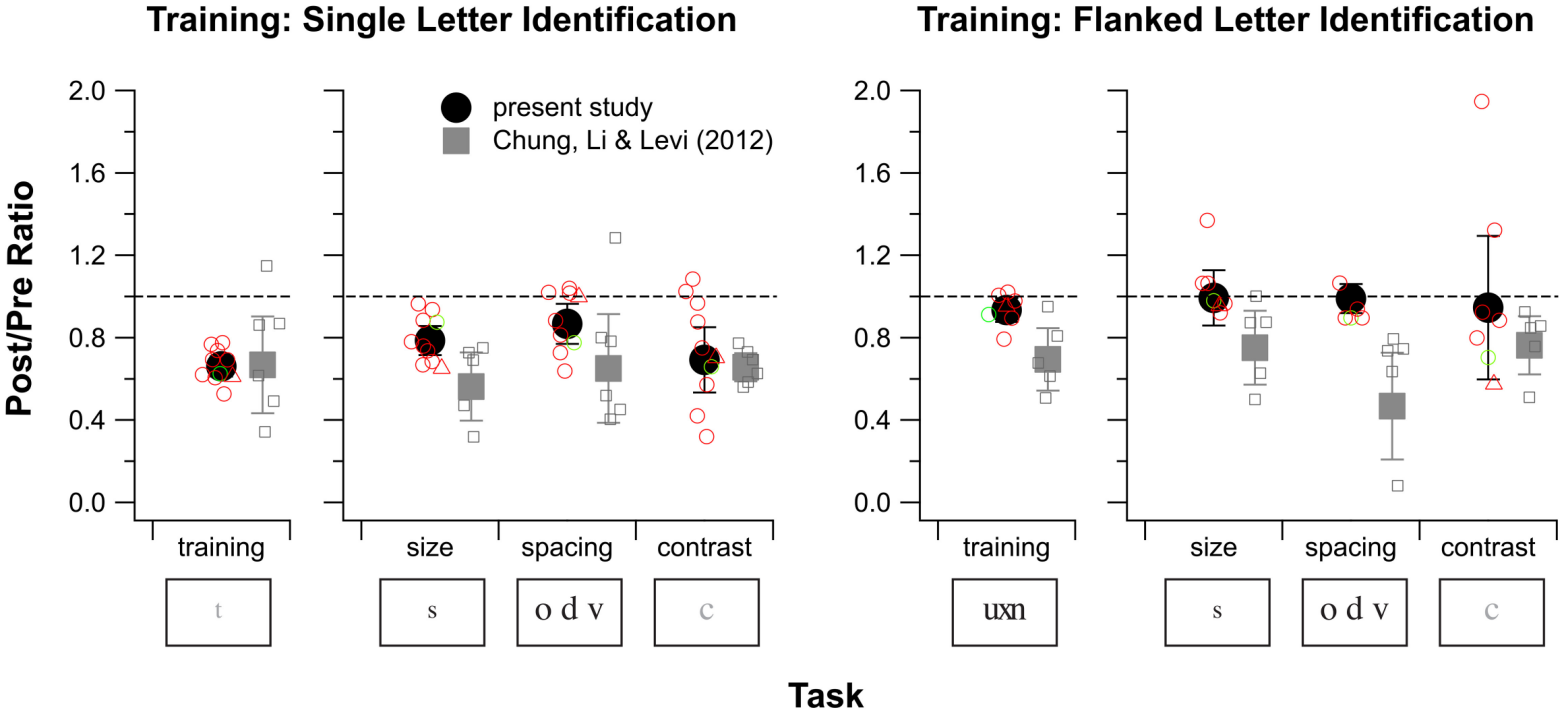
Training and transfer effects for single letter identification training **(left)** and flanked letter identification training **(right)** for observers S1–S10. For each of these training tasks, changes in performance for the training task are plotted in the left sub-panel and changes in performance for the three baseline tasks are plotted in the right sub-panel. Except for the values plotted for the training task for flanked letter identification, values plotted represent the Post/Pre threshold ratio (PPR) of the last and the first block of the training task (based on the fitted curves), or one of the three baseline tasks (size, spacing, and contrast threshold). For the training effect of flanked letter identification, Pre/Post ratios are used, because performance on this task was proportion correct rather than a threshold. A ratio of 1 (indicated by the dashed lines) represents no change, and ratios <1 indicate improved performance. Black solid circles and error bars are the mean and 1.96 × SEM from the present study. Gray squares and error bars are the mean and 1.96 × SEM, replotted from our previous study of training without donepezil (Chung et al., 2012). Open symbols represent individual observer data (red for amblyopes with strabismus and green for the amblyope without strabismus, circles for observers S1–S9, triangles for observer S10). For flanked letter identification, we were not able to measure the spacing limit for two observers during pretests, thus the number of open symbols is fewer than seven.

Next, we examined possible effects of donepezil on the magnitude of PL. The amount of learning was defined as the ratio of contrast thresholds between the 100th block and the 1st block of training trials, with values for these blocks derived from the fitted curve for each observer. The group average ratio as well as individual observers' ratios are plotted in the far left sub-panel in Figure 5. A *t*-test showed that the average ratio was significantly different from a value of 1 (*t*_df = 9_ = −13.15, *p* < 0.0001), indicating significant learning of single letter identification (mean ratio ± 1.96 × SEM: 0.66 ± 0.05). However, this average ratio was not significantly different from that obtained in our previous study (Chung et al., 2012), in which observers were trained without donepezil (mean ratio ± 1.96 × SEM: 0.67 ± 0.23; *t*_df = 5.45_ = −0.46, *p* = 0.67), indicating that donepezil did not substantially affect the magnitude of learning for the single letter identification task.

For the flanked letter task (Figure 2, right panels), there appears to be very little change in performance with training under donepezil. Therefore, we simply fit a linear function to describe each individual observer's set of data (the exponential function that we used for the single letter identification task resulted in a much poorer fit to the data, after accounting for the additional degrees of freedom). An improvement in performance due to training should yield a linear function with a positive slope that is significantly different from a slope of zero. For most observers, the slope of the linear function did not differ from zero (see *p*-values given in individual observers' plots), implying there was no improvement during training. When we compared the slopes (the rate of improvement) of the linear function of our group of observers with those from Chung et al. (2012), we found that the slopes were not significantly different between the two groups with or without donepezil (*t*_df = 4.33_ = −1.91, *p* = 0.12, see also Figure 3, right panel). However, the group-average data (Figure 4, right panel) appears to have a positive slope for the learning curve for the group without donepezil (plotted in gray) while the slope for the donepezil group is close to zero (plotted in red). The slope of learning for the no-donepezil group was an order of magnitude higher [0.0012 ± 0.0004 (mean ± 1.96 × SEM)] than that for the donepezil group (0.0002 ± 0.0004). Thus, donepezil does not seem to improve the rate of learning for the flanked letter identification task.

To assess possible drug effects on the magnitude of learning of flanked letter identification, we first assessed whether the magnitude of learning for the group of observers in this study differed from a value of 1 (no improvement), as we did for the single letter identification training task. Here, the amount of learning was defined as the ratio of proportion correct between the 1st block and the 100th block of training, based on the fitted linear functions. A *t*-test showed that the average ratio was not different from a value of 1 (*t*_df = 6_ = −2.14, *p* = 0.08) for the donepezil group. However, the ratio for this group was significantly different from that reported in our previous study in which donepezil was not administered (*t*_df = 5.19_ = 2.73, *p* = 0.04), indicating that donepezil had a deleterious effect on the magnitude of learning for the flanked letter identification task (Figure 5, right panel).

The absence of a significant improvement following flanked letter training under donepezil is puzzling, since it is in clear contrast to our previous study without donepezil [mean ratio ± 1.96 × SEM = 0.93 ± 0.06 (with donepezil) vs. 0.69 ± 0.15 (without donepezil)]. Also, following training using crowded acuity stimuli, Hussain et al. (2012) showed that both normal and amblyopic observers improved in crowded as well as single-letter acuities, thus confirming that crowding is amenable to training. We wondered whether the lack of improvement in the flanked letter training task was due to the fact that isolated letter performance had already substantially improved following Phase 1 training (Figure 2 and Figure S1), leaving no room for improvement for other letter identification tasks (although, as we shall see later, the absence of transfer from isolated letter training to the flanked letter baseline task suggested that there could be room to improve).

To address this possibility, we trained one amblyopic observer (S10, difference in crowded and uncrowded letter-chart acuity = 0.2 logMAR) in the reverse order—flanked letter task first (using the same sequence of drug administration, testing and training as for S1–S9), followed by single letter identification training, both while ingesting donepezil. This observer showed no significant improvement on the flanked letter task while taking donepezil (ratio between the 1st and the 100th block of trials = 0.95; see also Figure 2, third row, right column) but significant subsequent improvement on the single letter identification task (ratio between the 100th and the 1st block of trials = 0.62; see also Figure 2, third row, left panel), despite the fact that he had already performed roughly 10,000 trials of flanked letter training prior to single letter training.

This finding provides evidence against the possibility that observers training while taking donepezil were unable to improve on a second letter identification task because they had already reached their limit of improvement after the first letter identification task. Another possibility is that the sequential training procedure impairs learning on the flanked letter task, with or without donepezil. In our previous study (Chung et al., 2012), the two training tasks were performed using two separate groups of observers. In other words, it remains possible that learning of the flanked letter task is impaired when it is preceded by single letter identification training.

To test whether improvements on the flanked letter identification task (without donepezil) are possible in a sequential training design, we trained another amblyopic observer (S11) using a protocol identical to that of the original observers S1–S9—training with the single letter identification task, followed by a 3-week period of no training, then training with the flanked letter task, with the only difference being that S11 completed all pre- and post-tests and training *without* taking donepezil. This observer showed a modest improvement on the single letter identification task (ratio between the 100th and the 1st block of trials = 0.83), similar to two of the six observers in Chung et al. (2012) who showed very little improvement on this task. However, the slope of the linear fit of her data on the second training task was significantly different from zero (*p* = 0.001; ratio between the 1st and the 100th block of trials = 0.74, Figure S2). This significant learning of flanked-letter identification in the absence of donepezil in S11 suggests that the absence of improvement in the flanked letter task with training under donepezil seen in S1–S9 is not necessarily due to the sequential training paradigm.

### Transfer effects

Figure 5 summarizes performance data from baseline tasks, comparing pre- and post-data for single letter training, and pre- and post-data for flanked letter training. A Post/Pre threshold ratio (PPR) value of 1 (indicated by the dashed lines) represents no change in visual performance with training, and PPR < 1 indicates improved performance. For single letter training (Figure 5 left panel; red open circles are individual observer data; solid circles and error bars show the mean ± 1.96 × SEM), observers who were trained under donepezil showed significant improvements in each of the three baseline measures. These improvements were largest (PPR ≈ 0.7, a factor of about 1.4) for the contrast task, which is related to the training task, and smallest (PPR ≈ 0.9, a factor of about 1.1) for the spacing task.

Statistical testing showed that these PPR-values were all different from a value of 1 [*p*-values from *t*-tests: 0.0003 (acuity task), 0.040 (spacing task), and 0.0099 (contrast task)]. More importantly, none of these PPR-values were different from the corresponding values for the no-donepezil group [*p* = 0.073 (acuity task), 0.27 (spacing task), and 0.40 (contrast task)]. These findings for the baseline tasks further support the conclusion drawn from the direct training effects: that there is no detectable effect of donepezil on PL for the single letter identification task in people with amblyopia.

For flanked letter training under donepezil (Figure 5, right panel), observers did not show any improvement on the trained task, therefore it is not surprising that they also did not show an overall improvement in any of the three baseline measures. Indeed, *t*-tests showed that PPRs for all three baseline tasks were not significantly different from 1 for the donepezil group [*p* = 0.94 (acuity task), 0.85 (spacing task), and 0.91 (contrast task)]. However, although the PPRs for the three baseline tasks for the no-donepezil group (gray symbols) were all smaller than 1, none of them were different from the donepezil group PPRs [*p* = 0.078 (acuity task), 0.16 (spacing task), and 0.24 (contrast task)].

There is evidence that in amblyopic observers, the amount of improvement with PL depends on an individual's initial performance level (Li R. W. et al., 2008; Astle et al., 2013). Specifically, subjects with poorer initial performance showed greater improvement with training. In our study, initial performance level was comparable in the current group and that of the previous no-drug study for single letter training [contrast threshold 0.39 ± 0.17 (mean ± 1.96 × SEM) vs. 0.28 ± 0.12], and also for the flanked letter training groups (31.6 ± 13.6 percent-correct vs. 24 ± 12.8 percent-correct).

**Figure 6:**
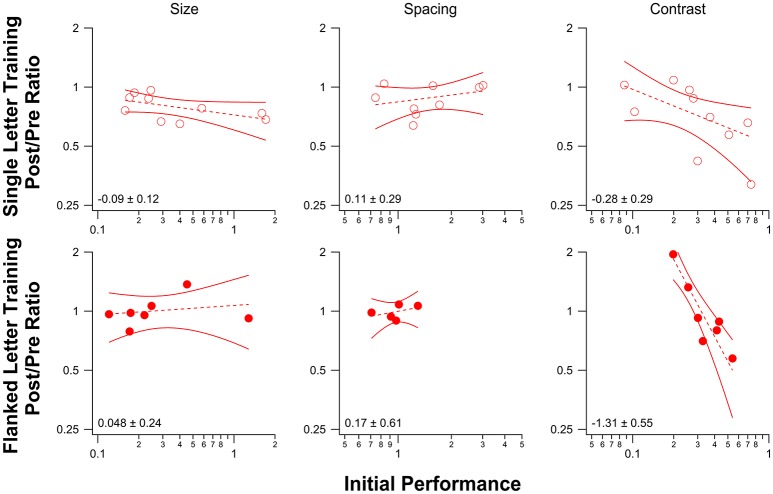
Post/Pre Ratios for each of the three baseline tasks are plotted as a function of initial performance for single letter identification training **(top)** and flanked letter identification training **(bottom)**. Each data point (red circle) represents the performance for one observer. Dashed lines represent linear regression lines for the data, with 95% confidence bands shown as solid red lines. The slope of each line (and 95% confidence interval) is displayed in the lower left corner in each plot. For the spacing measurement, the number of data points plotted is fewer than the number of observers for both training tasks because we were not able to obtain a reliable pre-test performance measure for some observers (the pre-test performance for all spacings except for the unflanked condition was poor, and we were therefore unable to fit a psychometric function to obtain a spacing threshold).

Figure 6 plots the improvement (PPR) vs. initial performance for observers in each of the three baseline tasks (single letter identification training—top row; flanked letter identification training—bottom row). We fit the data with power functions, which are plotted as straight lines on these log-log axes. The slopes (exponents and 1.96 × SEM) are shown in each panel. While there was no significant relationship between the amount of improvement and initial performance for size and spacing limits for either training task, contrast threshold for letter identification showed a strong negative correlation between the amount of improvement following flanked letter training (bottom right panel) and the initial performance, in line with previous work (Li R. W. et al., 2008; Astle et al., 2013).

## Discussion

Contrary to our hypothesis that increasing ACh levels would enhance PL in adults with amblyopia, we found that when a group of adults with amblyopia practiced a single letter identification task with concurrent administration of donepezil (a cholinesterase inhibitor), neither the magnitude nor the rate of learning was better than that obtained when training without donepezil. More importantly, donepezil seemed to halt learning of a task aimed at reducing crowding.

There are several important caveats that should be taken into account when interpreting our findings. First, given that this was only a pilot study, the sample size was rather small, but based on our sample size calculation, it was sufficient to detect statistically significant learning effects. However, our finding that donepezil had undetectable or even deleterious effects on PL makes it unlikely that it would be a useful adjunct to PL of these letter identification tasks in amblyopia.

Second, this study was not a randomized trial, and observers were aware that they were ingesting a drug, raising the possibility of placebo effects. A placebo effect would generally be expected to result in more rather than less improvement in the donepezil group, relative to the no-drug group, although it is also possible that negative expectations on the part of the observers regarding the drug (for example, anxiety about possible side effects) could have impaired performance and/or training. Future studies, especially those designed to assess possible clinical efficacy, should consider a randomized, double-masked design to avoid some of these issues.

A third concern may be that the no-drug and donepezil groups were not completely matched. They were reasonably well-matched for age (19–65 in the present study and 22–67 in the previous one; *p* = 0.92). However, the present donepezil study had a greater proportion of males (60%) than the previous one (27%) and a lower proportion of anisometropes (present: 10% vs. previous: 30%). Our previous study found no significant effect of amblyopia type. Visual acuity in the amblyopic eye (logMAR) was, on average, somewhat poorer in the current study [present: 0.62 ± 0.13 (SEM) vs. previous: 0.51 ± 0.11 (SEM)], because two of the observers had especially poor acuity. Interestingly, one of these observers (S5) showed significant improvement in single letter identification, while the other (S6) did not. However, excluding these two observers did not change the results.

Also, our observers were adults, well beyond the sensitive or critical period of visual development. Finally, in order to minimize any possible side effects, we used a low daily dose of donepezil (5 mg), corresponding to the lowest daily dose prescribed to patients with Alzheimer's disease. It is important to point out that our findings do not rule out a potential beneficial role for donepezil in neural plasticity in children with amblyopia or in adults with larger doses and/or longer periods of donepezil administration.

Recent findings from animal studies on the roles of neuromodulators in regulating brain plasticity, especially in visually-deprived rodents, have led to a great deal of interest in the possibility of enhancing treatment outcomes in humans with amblyopia (see Gore and Wu, 2016, for a recent review). In the present study, we chose to use donepezil, a cholinesterase inhibitor that is widely used in the treatment of Alzheimer's disease and is orally active and considered to be safe, with few side effects. Donepezil inhibits the metabolism of ACh in the synapse, thereby prolonging its effective lifetime and presumably augmenting whatever effects ACh would normally have at that synapse. Importantly, donepezil has been shown to enhance PL of a motion direction discrimination task in adult observers with normal vision (Rokem and Silver, 2010), with long lasting effects (Rokem and Silver, 2013), making it appear to be a strong candidate for enhancing PL in amblyopia. Donepezil also enhanced performance during PL on a three-dimensional object tracking task in subjects with normal vision (Chamoun et al., 2017).

To our dismay, our results suggest that combining donepezil with PL does not result in either more or faster learning of letter identification in adults with amblyopia, compared with the effects of PL alone. While there was clear PL of single-letter identification for observers taking donepezil during training, the group averages of the magnitude of improvement in each of the baseline tasks were no greater than what we observed without the drug in our previous study of adults with amblyopia (Figure 5). However, the group average data from single letter identification training show a different, more prolonged time course for the donepezil group, compared to the no-drug control group (Figure 4).

Our most unexpected finding is that training on flanked letter identification while ingesting donepezil resulted in very little PL. This is in clear contrast with our and other studies showing that PL with flanked targets can reduce crowding in adults with amblyopia (Chung et al., 2012; Hussain et al., 2012). In Chung et al. (2012), four of the five observers showed a significant improvement with flanked letter training. Similarly, in Hussain et al. (2012), eight of the ten observers showed a significant improvement following training with crowded acuity targets. It is not clear why observers failed to learn the flanked letter task while ingesting donepezil, but one possibility is that the mechanism for improvement in recognizing flanked targets is due to observers' learning how to more effectively ignore the flankers (Yashar et al., 2015; Zhu et al., 2016), consistent with the idea that learning reduces crowding by decreasing the size of a perceptual window that gathers relevant information from the stimuli (Sun et al., 2010). If so, the lack of an improvement when observers underwent flanked letter training suggests that donepezil may have impaired observers' ability to learn to ignore the flankers, and/or to optimally adjust the size of the perceptual window during task performance.

Previous work (Kosovicheva et al., 2012) showed that acutely increasing synaptic ACh levels by administering donepezil to adults had no effect on letter crowding in normal peripheral vision. However, here we demonstrate a failure to learn a specific task related to crowding—flanked letter identification—during cholinergic enhancement with donepezil. As shown in Figure 2, this is not because our observers trained on single letters first—reversing the order of training under donepezil for observer S10 resulted in no learning for the flanked letter task in the first phase of training but substantial learning of single letter identification contrast thresholds in the second phase.

Acetylcholine has multiple effects on visual cortical neurons and perception. For example, local administration of ACh reduces excitatory receptive field (RF) size in marmoset primary visual cortical neurons (Roberts et al., 2005), and systemic administration of donepezil decreases the spatial extent of the excitatory fMRI response to visual stimulation in human V1 (Silver et al., 2008). Acute administration of donepezil improves performance on a surround suppression task (Kosovicheva et al., 2012), perhaps because the reduction of excitatory RF size allows some V1 neurons to become more immune to the suppressive effects of the surround. In addition, donepezil sharpens the spatial tuning of visual perception (Gratton et al., 2017), a result that is also consistent with decreased excitatory RF size in visual cortex.

At the level of cortical circuits, ACh boosts feedforward inputs to cortex while suppressing lateral interactions within the cortex (layers 2/3 and 5). For example, in macaque V1, ACh enhanced response gain in the primary thalamocortical recipient layer 4c (Disney et al., 2007), but suppressed visual responses in 36% of recorded neurons outside this layer (Disney et al., 2012). We speculate that this increased intracortical suppression may be the reason that ACh not only does not reduce crowding (Kosovicheva et al., 2012), but may also inhibit the ability to learn to “uncrowd.”

While our amblyopic observers showed improved single letter identification when training under cholinergic enhancement, their improvement on average was no greater than a group that underwent PL in the absence of the drug. The observers who trained under donepezil also failed to show any learning or transfer to the subsequently trained flanked letter task, whereas for PL in the absence of donepezil, both learning and transfer were evident for training on the flanked letter task (Chung et al., 2012). This is especially unfortunate, since for many amblyopic individuals, particularly strabismic amblyopes, crowding is a major bottleneck for spatial vision and reading (Levi et al., 2007; Levi, 2008; Song et al., 2014).

Despite the fact that our study was motivated by the idea of enhancing plasticity in the amblyopic visual system, it also has implications for understanding the mechanisms that underlie PL. Currently much of the debate about the nature and mechanisms of PL is centered on whether the learning is specific or general and whether it takes place in early visual cortex and/or at higher levels (for recent reviews see Sagi, 2011; Watanabe and Sasaki, 2015). Physiological studies in non-human primates suggest that learning occurs primarily in higher-level decision areas rather than in lower level sensory areas—for motion direction discrimination, in area LIP but not in MT (Law and Gold, 2008, 2010). On the other hand, learning for some tasks has been shown to result in altered responses in visual areas as early as V1 (Schoups et al., 2001; Li W. et al., 2008) and V4 (Yang and Maunsell, 2004). Recent work in human adults with amblyopia suggests that PL primarily reflects high-level rule-based learning (Zhang et al., 2014).

Our results add a new level of complexity to these previous findings. Without donepezil, amblyopic observers can learn to identify both flanked letters and single letters, and PL of both of these tasks transfers to other tasks (Chung et al., 2012). However, with donepezil, adults with amblyopia show no evidence of learning or transfer for flanked letters. Importantly, it is not the case that they are unable to learn when cholinergic signaling is elevated. For single letter training, their improvement is similar in magnitude to that found without the drug. Thus, donepezil appears to selectively block learning of, and transfer to, flanked target identification. Understanding where and how donepezil acts to block learning and transfer under crowded conditions may provide a key to understanding the mechanisms of PL, as well as the crowding phenomenon itself.

## Ethics statement

This study was carried out in accordance with the recommendations of the Institutional Review Board of the Committee for the Protection of Human Subjects at the University of California, Berkeley, with written informed consent from all subjects. All subjects gave written informed consent in accordance with the Declaration of Helsinki.

## Author contributions

SC, RL, and DL designed the study; SC and RL prepared the experiment, RL tested the participants; SC and DL analyzed the data and prepared the figures. All authors contributed to the writing of the manuscript.

### Conflict of interest statement

The authors declare that the research was conducted in the absence of any commercial or financial relationships that could be construed as a potential conflict of interest. The reviewer BT and handling Editor declared their shared affiliation, and the handling Editor states that the process nevertheless met the standards of a fair and objective review.
