# Supplementary material for: Donepezil Does Not Enhance Perceptual Learning in Adults with Amblyopia: A Pilot Study
Source: Front Neurosci. 2017 Aug 7;11:448. doi: 10.3389/fnins.2017.00448 (PMC5545606; doi:10.3389/fnins.2017.00448)
Supplement: Supplementary file 1 [file Presentation1.PDF]

Supplementary Figures

Combining the cholinesterase inhibitor donepezil with perceptual learning in adults with amblyopia

Susana T.L. Chung, Roger W. Li, Michael A. Silver & Dennis M. Levi

School of Optometry, Vision Science Graduate Program, and Helen Wills Neuroscience Institute, University of California, Berkeley, CA 94720, USA

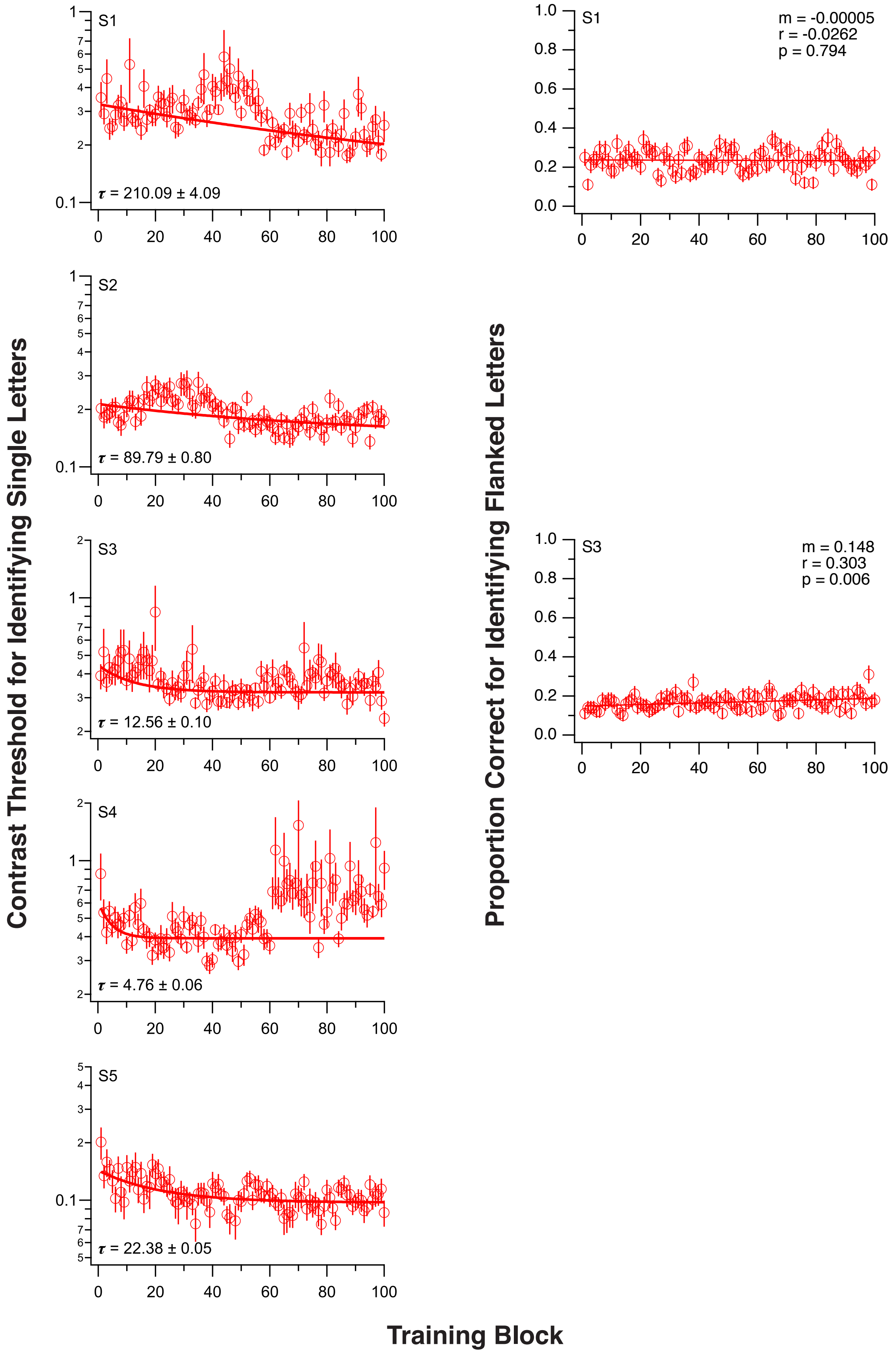
Contrast Threshold for Identifying Single Letters
S1
τ = 210.09 ± 4.09
S2
τ = 89.79 ± 0.80
S3
τ = 12.56 ± 0.10
S4
τ = 4.76 ± 0.06
S5
τ = 22.38 ± 0.05
Proportion Correct for Identifying Flanked Letters
S1
m = -0.00005
r = -0.0262
p = 0.794
S3
m = 0.148
r = 0.303
p = 0.006
Training Block

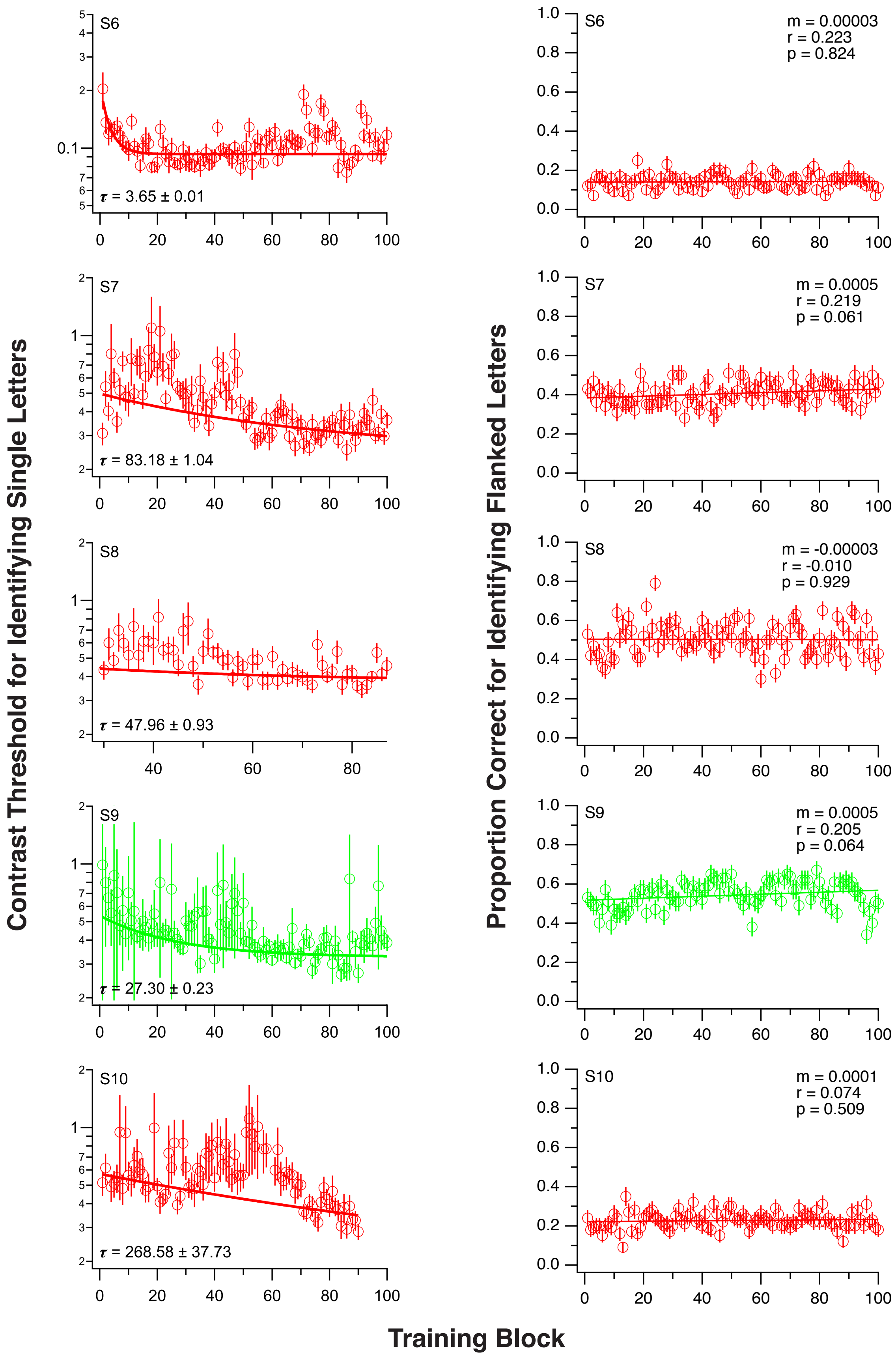

Contrast Threshold for Identifying Single Letters
Proportion Correct for Identifying Flanked Letters
Training Block
S6
τ = 3.65 ± 0.01
m = 0.00003
r = 0.223
p = 0.824
S7
τ = 83.18 ± 1.04
m = 0.0005
r = 0.219
p = 0.061
S8
τ = 47.96 ± 0.93
m = -0.00003
r = -0.010
p = 0.929
S9
τ = 27.30 ± 0.23
m = 0.0005
r = 0.205
p = 0.064
S10
τ = 268.58 ± 37.73
m = 0.0001
r = 0.074
p = 0.509

Figure S1. Training results from observers S1–S10. Performance for single letter identification task is shown on the left and performance for flanked letter identification task is shown on the right. Details of the plots are as in Figure 2 in the main text. Observers are color coded to represent the type of amblyopia: red representing strabismic amblyopia; green representing anisometropic amblyopia.

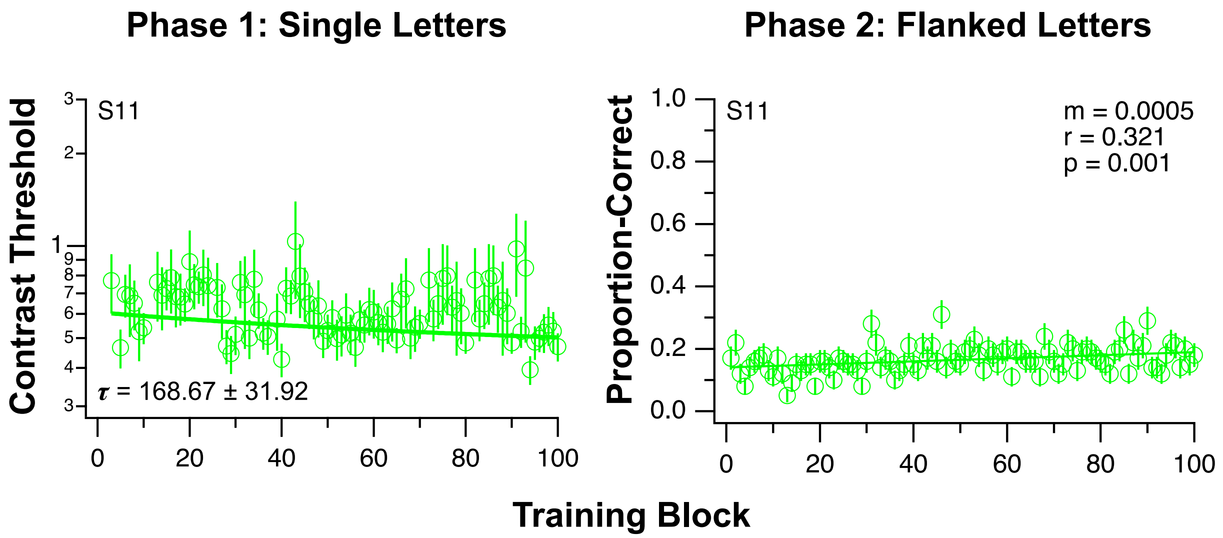

Figure S2. Training results from observer S11 who completed the two-phases sequential training *without* donepezil. Performance for single letter identification task is shown on the left and performance for flanked letter identification task is shown on the right. Details of the plots are as in Figure 2 in the main text.
